# Structural Alterations of the Social Brain: A Comparison between Schizophrenia and Autism

**DOI:** 10.1371/journal.pone.0106539

**Published:** 2014-09-04

**Authors:** Daniel Radeloff, Angela Ciaramidaro, Michael Siniatchkin, Daniela Hainz, Sabine Schlitt, Bernhard Weber, Fritz Poustka, Sven Bölte, Henrik Walter, Christine Margarete Freitag

**Affiliations:** 1 Department of Child and Adolescent Psychiatry, Psychosomatics and Psychotherapy, Johann Wolfgang Goethe-Universität, Frankfurt/Main, Frankfurt/Main,Germany; 2 Department of Psychiatry, Psychosomatics and Psychotherapy, Johann Wolfgang Goethe Universität Frankfurt/Main, Frankfurt/Main, Germany; 3 Department of Women’s and Children’s Health, Center of Neurodevelopmental Disorders at Karolinska Institutet (KIND), Stockholm, Sweden; 4 Division of Mind and Brain Research, Department of Psychiatry and Psychotherapy, Charité-Universitätsmedizin Berlin, Berlin, Germany; 5 Psychiatric University Clinics, University of Basel, Basel, Switzerland; The George Washington University, United States of America

## Abstract

Autism spectrum disorder and schizophrenia share a substantial number of etiologic and phenotypic characteristics. Still, no direct comparison of both disorders has been performed to identify differences and commonalities in brain structure. In this voxel based morphometry study, 34 patients with autism spectrum disorder, 21 patients with schizophrenia and 26 typically developed control subjects were included to identify global and regional brain volume alterations. No global gray matter or white matter differences were found between groups. In regional data, patients with autism spectrum disorder compared to typically developed control subjects showed smaller gray matter volume in the amygdala, insula, and anterior medial prefrontal cortex. Compared to patients with schizophrenia, patients with autism spectrum disorder displayed smaller gray matter volume in the left insula. Disorder specific positive correlations were found between mentalizing ability and left amygdala volume in autism spectrum disorder, and hallucinatory behavior and insula volume in schizophrenia. Results suggest the involvement of social brain areas in both disorders. Further studies are needed to replicate these findings and to quantify the amount of distinct and overlapping neural correlates in autism spectrum disorder and schizophrenia.

## Introduction

Autism spectrum disorder (ASD) and schizophrenia (SCZ) are biologically based psychiatric disorders that share a substantial number of etiologic factors and phenotypic characteristics. For instance, rare and partly overlapping copy number variants have been identified to be a strong genetic risk factor for both disorders [1], and relatives of individuals with ASD are more likely to have a family history of SCZ [2].

Both disorders are influenced by deficits of the social brain [2], [3], a specialized neural network dedicated to social cognition comprising in particular the medial prefrontal cortex (MPFC), the posterior temporal sulcus, and the adjacent temporo-parietal junction, the anterior cingulate cortex (ACC), the insula, the amygdala, the inferior frontal gyrus, and the interparietal sulcus [4], [5]. Social cognition refers to psychological processes that benefit social exchanges, in particular, a specific cognitive ability, called “Theory of mind” (ToM) or mentalizing, allows humans to explain and predict the behavior of conspecifics by inferring their mental states [6]. FMRI studies have shown aberrant activation in SCZ and ASD using mentalizing and basic emotional tasks. In SCZ aberrant neural activation in fronto-temporo-parietal regions and in amygdala were found [7]–[13]. Also, in ASD reduced activation in regions of the social brain during processing of social information has been described in the right pSTS, amygdala and fusiform gyrus [14]–[21].

Moreover, in both disorders abnormalities in global brain volume measures have been reported when compared to typically developing subjects (TD). In ASD, a greater total brain volume is present mainly in early childhood, rarely in adults [22]. In SCZ, smaller global GM and WM volumes have been reported in meta-analytic studies [23]–[25]. Meta-analyses of voxel-based morphometry (VBM) studies reported volume alterations of social brain areas in both disorders. In ASD smaller grey matter (GM) volumes were found in the temporal lobe, MPFC, amygdala/hippocampus, and precuneus [26], , instead larger GM volumes have been reported in the lateral prefrontal cortex and temporo-occipital regions [26], [22], [27]. Structural alterations in ASD seem to be age-related, as in adults with ASD structural alterations in fusiform gyrus, cingulum, amygdala and insula were less often reported compared to children/adolescents [22], [27], [1]. Similar to ASD, in SCZ structural alterations have been found in the social brain and meta-analyses have described smaller GM volumes in fronto-temporal regions, ACC, hippocampus/amygdala, and the insula [28]–[31], [23]. GM alterations were more extensive in patients with long illness duration possibly indicating a neurodegenerative process [29], [30], [32], [28]. A recent meta-analysis implemented anatomical likelihood estimation (ALE) to compare VBM studies on both SCZ and ASD [33]. Lower GM volumes in the limbic-striato-thalamic circuitry compared to controls were found as a structural overlap between SCZ and ASD. Distinct volumetric alterations were observed in the amygdala, caudate, frontal, and medial gyrus (SCZ) and putamen (ASD).

In summary, studies on structural alterations in ASD and SCZ compared to TD have shown GM volume alteration in social brain areas with a high diversity of brain volume changes within the social brain network, which are in part contradictory. The contradictions may be explained by the phenotypic differences within disorders, age and IQ effects, or by different methods used for data acquisition and analysis [32], [31], [27]. Therefore, a direct comparison of both disorders within a unitary methodological framework is necessary to clearly describe overlapping and disorder specific alterations of brain volume [34]. In the present VBM-study, ASD, SCZ, and TD were compared directly with respect to global and regional brain volume. Moreover, structural alterations were correlated with differences in mentalizing abilities and disorder specific symptom severity.

This study aims to address three hypotheses: First, in global brain measures, a slightly lower total GM and total white matter volume is expected in SCZ compared to TD. No differences in global brain measures are expected between ASD and TD, as greater total GM volume was found mainly in ASD children, but not in adults. Second, we hypothesize that in both disorders compared to TD, lower GM volumes are present social brain areas, reflecting impairments in social cognition. As ASD and SCZ show different and in part contrary deficits in social cognition (i.e. in ASD the ability to attribute mental states to others is deficient, while in paranoid SCZ these attributions are intensified), we expect distinct volume alterations comparing SCZ with ASD. Third, associations between the extent of volume alterations in these areas and behavioral data (symptom severity and mentalizing abilities) are expected.

## Methods

### Participants

Thirty-four patients with ASD (3 females, age range 14 to 33 years, mean age 19.06, SD 5.12; mean IQ 105.73, SD 12.92), 21 patients with SCZ (5 females, aged 14 to 33 years, mean age 24.67, SD 5.20; mean IQ 103.33, SD 11.21), and 26 TD (4 females, aged 14 to 27 years, mean age 19.54, SD 3.46; mean IQ 107.75, SD 11.97) were investigated. The ASD sample consisted of 16 patients with Asperger Syndrome, 11 patients with childhood autism and 7 with atypical autism. The study was approved by the local ethical committee of the medical faculty, JW Goethe-University, Frankfurt am Main, and was carried out according to the declaration of Helsinki. All subjects (and their parents in case of underage subjects) gave their written informed consent for participation in the study.

The following inclusion criteria were met by the clinical groups: 1) diagnoses according to ICD-10 (F84.0, F84.1, F84.5; F20.0); 2) no additional neurological or chronic medical disorder; 3) no other comorbid psychiatric disorder; 4) IQ >70. The diagnosis of paranoid hallucinatory schizophrenia (ICD-10: F20.0) was done by thorough clinical examination and validation by at least 2 experienced psychiatrists. The diagnoses of childhood autism, Asperger Syndrome or atypical autism (ICD-10: F84.0, F84.1, F84.5) was additionally confirmed by the Autism Diagnostic Interview - Revised (ADI-R, German version; [35], [36]) and Autism Diagnostic Observation Schedule (ADOS, German version; [37], [38]). In order to provide additional information on positive and negative symptoms, the Positive and Negative Syndrome Scale (PANSS, [39]) was obtained from SCZ. In SCZ, mean duration of illness was 67 months (SD: 45 months), ranging from 2 months to 144 months. Given the age at examination and the illness duration, 40% to 50% of the patients were considered as adolescent onset schizophrenia and 50% to 60% as adult onset schizophrenia (missing data in 2 datasets).

To exclude additional psychiatric disorders, all subjects were explored by experienced psychiatrists. In addition, participants filled in the youth or young adult self-report (YASR/YSR; [40], [41]), a screening instrument to assess self-rated psychopathology. Also, the child behavior checklist/young adult behavior checklist (CBCL/YABCL; [42], [41]) was completed by a parent, where possible. All TD subjects showed YASR/YSR and CBCL/YABCL subscales T-scores <67 (below borderline clinical cut-off). SCZ were older than ASD and TD. Therefore, age was controlled as a covariate in all further analyses.

### Psychological assessment

IQ was measured by the Raven Standard Progressive Matrices (SPM, [43]). Handedness was determined by the Edinburgh handedness inventory [44]. To characterize deficits in mentalizing abilities, all subjects were investigated using the ‘reading the mind in the eyes’ test (RME) [45]. In this test, participants were asked to identify complex emotional and non-affective states in images presenting human eyes.

Demographic data, clinical characteristics, and dosage of daily neuroleptic treatment chlorpromazine equivalent doses according to Woods [46] are shown in Table 1. Within the SCZ sample, 21 patients were treated with at least one of the following substances: Risperidone/Paliperidone (7), Quetiapine (5), Olanzapine (2), Clozapine (4), Aripiprazole (2), Haloperidol (1), Benperidol (2), Chlorprothixen (2); Escitalopram (1), Fluvoxamine (1), Ziprasidone (1); (missing data in 2 datasets); within the ASD sample 7 patients were treated with at least one of the following substances: Risperidone (2); Methylphenidate (5); Sertraline (2), Escitalopram (1).

**Table 1:** Sociodemographical and clinical parameters of ASD, SCZ and TD.

Parameter		ASD	SCZ	TD	P<0.05
		(N = 34)	(N = 21)	(N = 26)	
		Mean (SD)	Mean (SD)	Mean (SD)	
age [years]		19.06 (5.12)	24.67 (5.20)	19.54 (3.46)	SCZ>TD,ASD
gender (m/f)		31/3	16/5	22/4	–
IQ		105.73 (12.92)	103.33 (11.21)	107.75 (11.97)	–
PANSS^a^	Ps	–	14.43 (5.35)	–	
	Ns	–	17.33 (6.03)	–	
	Gps	–	30.90 (9.95)	–	
	T	–	62.67 (17.74)	–	
ADOS^b^	C	3.65 (1.41)	–	–	
	Si	7.76 (2.84)	–	–	
	T	11.41 (3.82)	–	–	
ADI-R^c^	Si	21.96 (5.96)	–	–	
	Cl	14.04 (4.61)	–	–	
	Rb	5.87 (2.50)	–	–	
RME^d^		16.74 (4.25)	20.57 (2.27)	21.15 (2.74)	TD, SCZ>ASD
Medication (mean CPZ-eq [mg])^e^		300 (N = 2)	478.7 (367.6) (N = 15)	–	

Significance threshold was defined as P<0.05.

a PANSS = Positive and Negative Syndrome Scale; p = positive scale; ns = negative scale; gps = general psychopathology scale; t = total;

b ADOS = Autism Diagnostic Observery Scale; missing data in 2 cases; c = communication; si = social interaction; t = total;

c ADI-R = Autism Diagnostic Interview - Revised; missing data in 2 cases; si = social interaction; cl = communication and language; rb = restricted and repetitive behaviours;

d RME = ’Reading the Mind in the Eyes’ test,

e CPZ-eq = Chlorpromazine equivalents.

### Data acquisition and voxel-based-morphometry analysis

Structural magnetic resonance images were acquired using a 3 Tesla Siemens Allegra scanner (Erlangen, Germany) with a 1-channel head coil. Data were recorded using a T1-weighted MDEFT-sequence [47] with parameters as follows: TR: 10.55 ms, TE: 3.06 ms; TI: 680 ms; flip angle: 22°. One dataset consisted of 176 axial images with an inplane resolution of 1 mm^3^, field of view: 256 mm; slice thickness: 1 mm.

### Imaging data pre-processing

All datasets were manually reviewed for head motion and image quality. Datasets with low image quality or motion artefacts were excluded from analysis. Structural images underwent pre-processing using optimized VBM as implemented in SPM8 (http://www.fil.ion.ucl.ac.uk/spm/software/spm8) according to the standardized procedure [48]. Images were segmented into GM and white matter (WM). Global GM, global WM, and total brain volume (TBV) were calculated. Subsequently, images were normalized by creating a DARTEL-template to provide an increased accuracy of inter-subject alignment. The images were resampled to 1.5×1.5×1.5 mm^3^ voxel size. Images were smoothed (using an 8 mm FWHM isotropic Gaussian kernel) and normalized to MNI space. Data were corrected for differences in global GM/WM volume. As treatment with neuroleptic medication differed between groups, daily chlorpromazine-equivalents were tested for association with local volume alterations in SCZ.

### Statistical analysis

Total tissue volumes were calculated by summing the partial volume estimates multiplied by the voxel volume across the entire brain. Between-group differences in global brain measures were examined using an ANOVA with factor group including age as a covariate. Subsequent comparisons between means were performed using Bonferroni’s post hoc test. Individual GM and WM segments were subjected to a voxel-wise multiple regression analysis. The differential effects of the three groups (ASD, SCZ and TD) were assessed within an ANCOVA model including age and global GM or global WM volume as covariates. ANCOVA was followed by between group comparisons. Significance threshold was set at P<0.001, uncorrected (K>20). Anatomical regions and denominations are reported according to the atlas of Talairach [49], [50]. Coordinates are provided as maxima in given clusters according to the standard MNI-template. To identify brain abnormalities in ASD und SCZ associated with illness severity and social cognition, individual peak voxel data were extracted from the regions resulting from between groups comparison and associated with RME, ADOS, and PANSS using Pearson’s correlation. Results reported as significant were defined as P<0.05 and r>0.4. Data were analysed using SPSS Statistics version 17.0.

## Results

### Global brain measures

**Figure 1:**
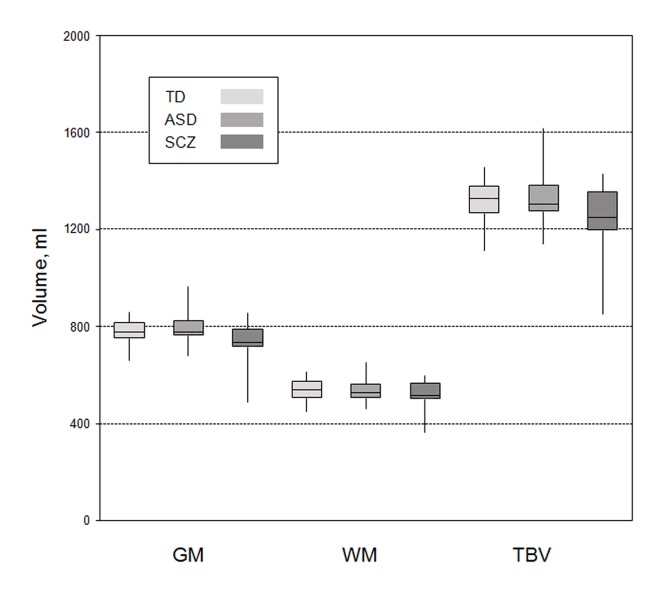
Box plots of global brain measures. Box plots are given for autism spectrum disorder (ASD), schizophrenia (SCZ) and typically developed control subjects (TD) each with global gray matter (GM), global white matter (WM) and total brain volume (TBV). The horizontal line in each box indicates the median, while the top and bottom borders of the box mark the 25th and 75th percentile, respectively. The vertical lines above and below the box mark the range of distribution.

Global brain measures were calculated in ASD (global GM [mean, SD]: 790.26 ml, 52.32 ml; global WM: 535.93 ml, 41.28 ml; TBV: 1326.19 ml, 94.87 ml), SCZ (global GM [mean, SD]: 731.69 ml, 85.63 ml; global WM: 518.96 ml, 59.08 ml; TBV: 1250.65 ml, 142.79 ml), and TD (global GM [mean, SD]: 777.48 ml, 52.31 ml; global WM: 537.50 ml, 42.53 ml; TBV: 1314.98 ml, 92.43 ml). No differences were found between groups in global GM (F2,77 = 2.365; P = 0.101), WM (F2,77 = 0.894; P = 0.413) or TBV (F2,77 = 1.640; P = 0.201) (Figure 1).

### Voxel based morphometry

**Figure 2:**
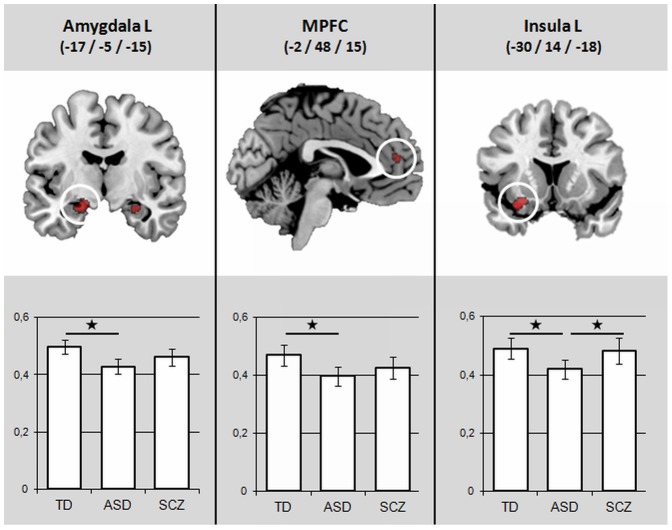
Results of conjunctional VBM analysis of T contrasts. All statistical parametric maps were thresholded at P<0.001 with a cluster level K>20, age was added as a covariate. In column diagrams, beta-values are given for each cluster. Significant contrasts (P<0.05) are marked with star (*). *Left:* contrast TD>ASD in a coronal view (Y = −5); *Middle:* contrast TD>ASD in a saggital view (X = −2); *Right:* contrast SCZ>ASD in a coronal view (Y = 14).

As results did not survive a FWE/FDR correction, they are given on a P<0.001 level, uncorrected. A main effect of group was observed for GM volume in the left anterior insula (AI), left amygdala and occipital medial area (Table 2). No main effect of group was found for WM volume. Thus, between groups analysis was performed for GM only. In the contrast SCZ vs. ASD, GM volume in left AI was smaller in ASD (Figure 2). In the contrast ASD vs. TD, smaller GM volumes were found in ASD in left amygdala, left AI and additionally in the anterior MPFC and right amygdala (Figure 2). In the medial occipital area, GM volume was larger in ASD. The contrast SCZ vs. TD did not reveal any significant GM alterations.

**Table 2:** Summary of main effect results and between group analyses in MNI space on a p<0.001 level.

Brain region	MNI Coordinates			Z-score	Cluster size
	x	Y	z		[voxels]
**main effects**					
Insula L	−30	14	−18	3.98	88
Amygdala L	−17	−5	−15	3.58	72
Occipital medial area	38	−74	23	3.43	29
**TD>ASD**					
Amygdala L	−17	−5	−15	4.10	319
Amygdala R	27	−9	−18	3.37	39
MPFC	−2	48	15	3.56	28
Insula L	−30	15	−18	3.54	22
**ASD>TD**					
Occipital medial area	39	−75	23	3.95	118
**SCZ>ASD**					
Insula L	−30	14	−18	4.22	224

Antipsychotic treatment must be considered as a potential bias on regional brain volume. No association was observed with amygdala, insula, or MPFC volume and chlorpromazine-equivalents.

### Correlations between volumetric and behavioral data

**Figure 3:**
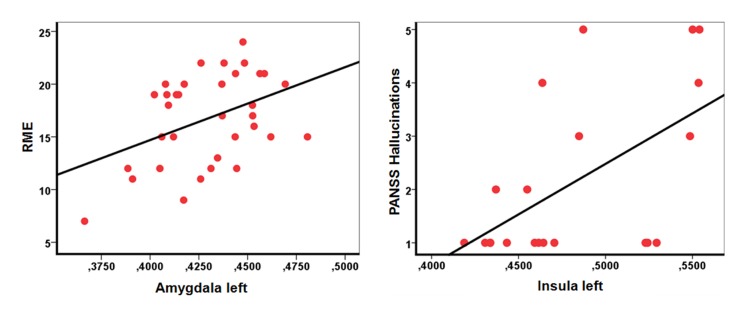
Correlation plots. Left: Correlation between left amygdala GM volume (beta-values) and the ‘reading the mind in the eyes’-score (RME) in autism spectrum disorder (r = 0.41; P = 0.015). The RME-score represents the number of correctly identified emotions of pictures depicting facial expressions (maximum score = 28). Right: Correlation between left insular GM volume (beta-values) and the PANSS hallucinatory behaviour score in schizophrenia (r = 0.56; P = 0.008). The hallucinatory behaviour score represents the degree of a patient’s verbal report or behaviour indicating perceptions which are not generated by external stimuli. The score ranges between 1 (absent) and 7 (extreme).

In SCZ, a positive correlation was found between left AI GM volume and the PANSS hallucinatory behavior score (r = 0.56; P = 0.008). No correlation was found between GM alterations and illness duration. In ASD, a positive correlation was found between left amygdala volume and the RME-score (r = 0.41; P = 0.015) (Figure 3). As one peripheral data point (Figure 3, data point (0.37/7)) was suspect being an outlier, a second analysis was performed with the remaining data. The correlation persisted on a trend level.

## Discussion

To our knowledge, this is the first structural MRI study that compared ASD and SCZ directly. The following main findings were revealed: 1) No global GM or WM alterations were present between groups. 2) In VBM analysis, GM alterations were present mainly in regions associated with social cognition (amygdala, MPFC and insula). Direct comparison of both disorders demonstrated a smaller GM volume in the left anterior Insula (AI) in ASD compared to SCZ. ASD showed smaller local GM volumes compared to TD in the bilateral amygdala, left AI and anterior MPFC. No differences were found between SCZ and TD. 3) Alterations in GM volume correlated significantly with specific parameters of psychopathology and social abilities: hallucinatory behavior with AI volume in SCZ, and mentalizing abilities with amygdala volume in ASD.

### Global brain measures

No alterations in global GM/WM were found between groups. Although meta-analytic studies in SCZ reported alterations in global brain parameters such as smaller global GM, global WM and TBV [23], [51], [52] original data reported contradicting results. Out of 32 cross-sectional studies, that were included in meta-analytic studies on first episode SCZ [24], [53], only 7 studies reported significant results. As alterations are subtle [54], large samples are needed to detect these differences. Thus, it is not surprising, that in our study, no differences between groups regarding global brain measures were detected. In ASD compared to TD, previous studies reported larger GM, WM and TBV predominantly in children, but not in the adult population [22]. This is in accordance with results of this study.

### Voxel based morphometry

#### 1. Direct comparison of ASD and SCZ

This study suggests a dysfunctional involvement of the AI in both disorders. ASD patients showed a smaller GM volume in the left AI compared to SCZ, whereas a positive correlation was found between insular GM volume in SCZ and PANSS hallucinatory behavior score.

In ASD and SCZ, the insula was consistently affected in structural and functional MRI studies [30], [55]–[57], [29], [58], [28]. Similar, smaller insular GM volumes were found in SCZ and ASD in a study using an anatomical likelihood estimation (ALE) approach [33].

The AI is an extensively connected, multifaceted brain region that is involved in numerous brain functions [59]. This brain region is highly involved in processing sensory stimuli with a unique role in interoception, monitoring the physiological reaction like heartbeat frequency, skin conductance, pain and touch [60]–[63] and also the emotional component of interoceptive awareness [64], [65]. The interceptive awareness of the body as an independent entity that is distinct from external environment, is a precondition for self and non-self discrimination [66], [59]. Furthermore, AI has been implicated in empathic abilities [67], [68], [62] and is involved in auditory and facial affect processing [69]–[71]. One theory, the salience network hypothesis [72]–[74] conceptualizes the disparate AI functions using a comprehensive “network perspective”. According to this model, the AI is the integral hub of the salience network, and represents a multimodal salience detector that identifies the most relevant among several internal and external stimuli. It is associated with segregate functions (i.e. processing of sensory information like pain, social cues like facial expressions, and the analysis of own emotional state) which all facilitate subjectively relevant information.

A salience network dysfunction has been proposed to play a major role in ASD and SCZ [72], [75]–[78]. In ASD the smaller insular GM volume found in this study fits well with AI hypo-activity reported by fMRI studies on tasks of social cognition [58]. A structural insular abnormality might lead to a disconnection between the AI and sensory and limbic structures, resulting in limited ability to identify salient stimuli necessary for adapting adequately to the social environment [72]. Due to this dysfunction, social cues might not be identified as salient and thus not labelled as emotionally rewarding in the insula. In contrast, sensory stimuli might be considered as salient, which may underlie sensory interests, repetitive behavior, or even anxiety and avoidance in ASD.

In SCZ, brain regions underlying the salience network are consistently changed, in example the insular cortex [79], [29], [80], [77]. Studies on high-risk individuals show, that smaller GM volume of ACC and Insula, both major regions of the salience network, are associated with transition to psychosis [81], [82]. Salience network dysfunction has been proposed to play a key role in positive and negative symptoms in SCZ [75], [83], [77]. In this study, we found a significant relation between insula volume and PANSS hallucinatory behavior score. Hallucinations can be conceptionalized as a failure to differentiate an internally generated from an externally sensory experience [84], [85]. Functional and structural studies revealed that the insula, along with regions traditionally known as language areas, seem to play a key role in auditory hallucinations in SCZ [86]–[92]: A Insula dysfunction might cause a confusion of the two sources, resulting in internal sensory information being attributed to external sources [93]. Audible thoughts, thought insertions, and hallucinations might be the consequence. Still, a positive correlation between insula volume and PANSS hallucinatory behaviour score was surprising as it stays in contrast to reports of insular GM volume loss in SCZ [29]. Further studies are needed to investigate significance of these alterations in more detail and to resolve the mentioned inconsistencies.

#### 2. Direct comparison of ASD and TD

The ASD group was characterized by significantly smaller GM volumes in bilateral amygdala, left AI and anterior MPFC compared to TD. Hence, only areas involved in social cognition were significantly smaller. Smaller GM volume in the amygdala-hippocampal complex represents a common finding in VBM-studies in ASD [53], [94]–[97]. The amygdala is involved in emotion recognition and affective Theory of Mind and is thus a key player in social cognition [98]. The association of social impairment and abnormalities of the amygdala have been reported across many different scientific fields [99]–[101]. In a meta-analysis of functional neuroimaging studies in ASD, hypoactivation of the amygdala has been found in different social cognitive tasks [102], [103]. Because a smaller cortical GM volume has been frequently associated with a reduced function in the affected structure [104], the smaller GM volume in the amygdala in ASD fits well to the lower amygdala activation in functional neuroimaging studies. This is supported by the positive correlation we found between mentalizing abilities and amygdala volume in ASD.

In agreement with results of this study, Mc Alonan found a significantly smaller insular GM volume in ASD [105]. Greater insular surface area is associated with poorer social behaviour in ASD [106] and a meta-analysis of functional MRI studies examining social processing identified the AI as a consistent locus of hypo-activity in autism [58]. Altered functional connectivity of AI with Amygdala and somatosensory regions were also present in ASD [107]. These structural and functional findings could underlie typical ASD symptoms like altered emotional experiences and impaired social abilities.

In MPFC, we found a smaller GM volume in ASD. According to a functional division of the MPFC this area is located in the anterior MPFC [108]. The MPFC, in particular its anterior part, is a key region of mentalizing abilities. It is involved during communicative intention [109]–[112] and triadic interactions [113]{Amodio 2006 #141 [114], [115]. Different lines of evidence imply alterations of MPFC in ASD. For example, injuries of the MPFC have led to deficient mentalizing abilities and autism-like behavior [116]; histologic abnormalities in this region have been found in animal models of autism [117], [118], and in postmortem-studies of ASD patients [119]. Different neuroimaging techniques have revealed a reduced activity in the MPFC in ASD patients using MEG [120], PET [121], and functional MRI during Theory of Mind tasks [122], [14]. The reduced activity in the MPFC corresponds well with the smaller GM volume across ages described in previous meta-analyses [27]. This study provides an additional confirmation for this finding.

#### 3. Direct comparison of SCZ and TD

No structural alterations were found in SCZ compared to TD. This is in disagreement with our hypothesis, as brain volume alterations were expected in areas encoding social cognition in SCZ. Smaller regional brain volumes were consistently reported in meta-analyses [55], [31], [29]. However, these results are highly heterogeneous, as age of onset and illness duration influence results of VBM analyses causing different alterations in regional GM volume [28], [31]. In this study, three aspects might explain the lack of observed differences between SCZ and TD: First, SCZ comprised the smallest group of participants. Thus, the study has lacked power to find smaller volumetric changes [123], which must be considered as a limitation. Second, with a mean age of 24.7 years (range 14 to 33 years) the SCZ patients in our study were relatively young. In SCZ, GM alterations are more pronounced in samples with rising illness duration and age [51]. Third, the examined population was a mixed sample of adolescent-onset SCZ and adult-onset SCZ. A heterogeneous sample in terms of age of onset might have contributed to a lack of alterations in GM volume in this contrast.

### Study limitations

Some aspects must be considered as limitations of this study. (1) The study was designed to detect only large volume differences; due to limited sample size and a lack of power, possibly existing smaller volume alterations were not detected. We can therefore only interpret the differences detected in our study but not the lack of differences. Results need to be considered preliminary, as they were reported given on a p<0.001 level and did not withstand a more stringent and conservative correction for multiple comparisons across the brain. (2) The SCZ sample was heterogeneous in regards of disease onset comprising both adolescent and adult onset schizophrenia.

## Conclusion

In summary, this study compared ASD and SCZ patients directly for global and regional alterations in brain structure. Results emphasize distinct brain substrate in both disorders, as GM alterations of the social brain were prominent in ASD only. Still, we found that disturbances in the insula may play an important role in both conditions. As a morphologic correlate, this study revealed a lower insular GM volume in ASD; in SCZ individuals, a positive correlation of insular GM volume and PANSS hallucinatory behavior score was found, which contrasts to smaller insular volume frequently reported in SCZ. Further studies are needed to replicate the described findings and investigate neurophysiological significance of these alterations for ASD and SCZ in more detail.
